# Supplementary material for: Simultaneous Analysis of the p16 Gene and Protein in Canine Lymphoma Cells and Their Correlation with pRb Phosphorylation
Source: Vet Sci. 2022 Jul 29;9(8):393. doi: 10.3390/vetsci9080393 (PMC9416461; doi:10.3390/vetsci9080393)
Supplement: Supplementary file 1 [file vetsci-09-00393-s001.zip › vetsci-1826278-supplementary.pdf]

Supplementary Materials

(a)

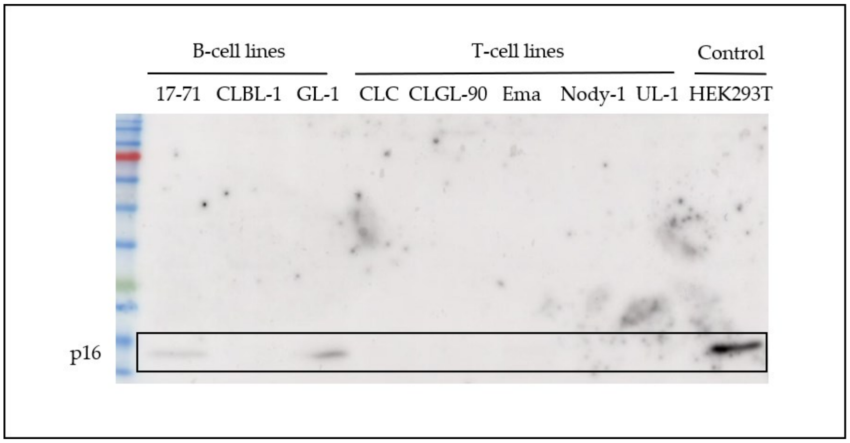

(b)

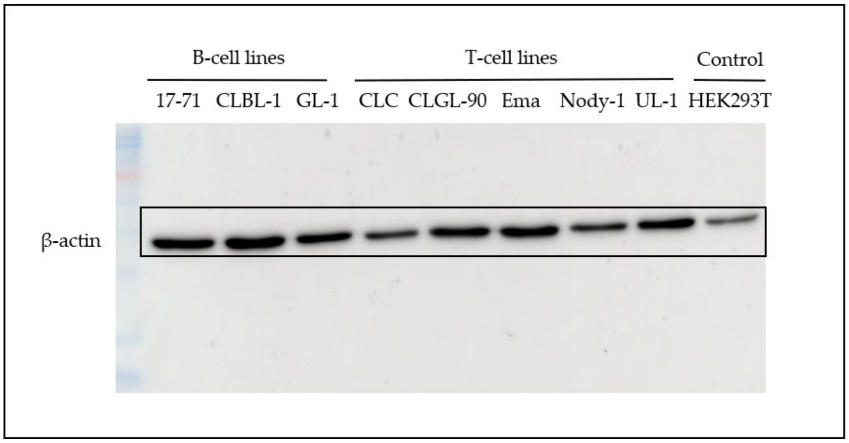

Figure S1. HEK293T human cell line was used as a positive control of p16 protein expression. The expression of the p16 protein in canine lymphoma and leukemia cell lines (a); β-actin was used as the endogenous control (b). The protein expression was assessed using the western blot analysis.

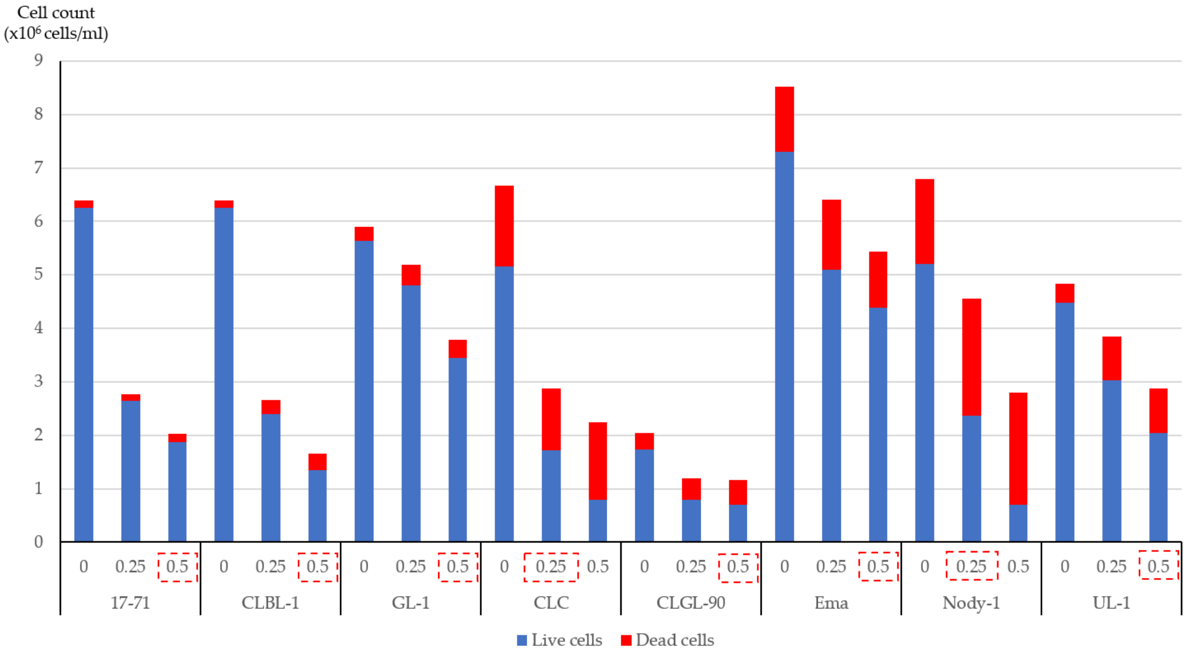

Figure S2. Cell viability of canine lymphoma and leukemia cell lines after treated with 5-Aza (0.25 μM and 0.5 μM) and without it (0 μM). After treated with 5-Aza for 72h, total live cells equal to or more than 50% was considered for western blots analysis and real-time PCR [22].

(a)

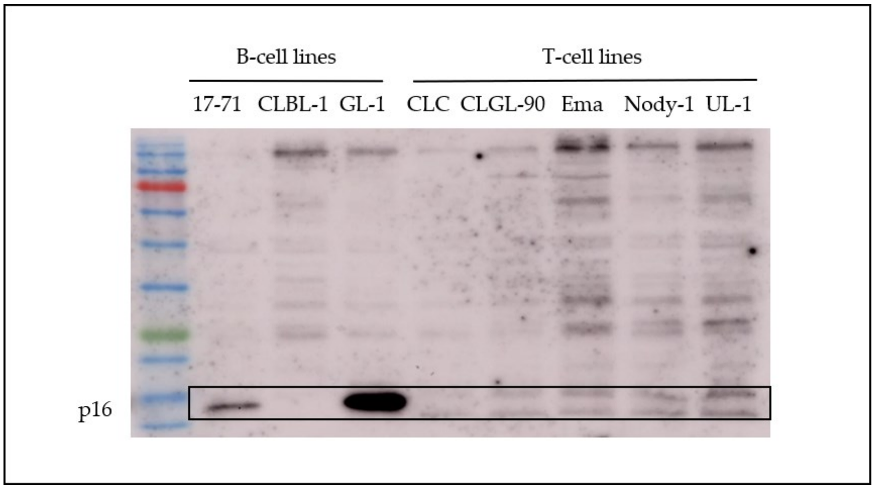

(b)

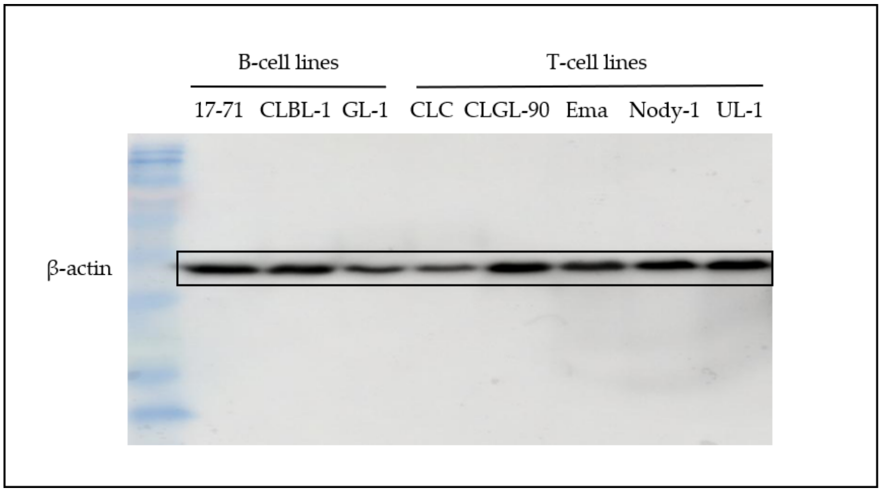

Figure S3. Original images for Figure 1. The expression analysis of the p16 protein in canine lymphoma cell lines (a); β-actin as endogenous control (b). The protein expression was assessed using western blot analysis.

(a)

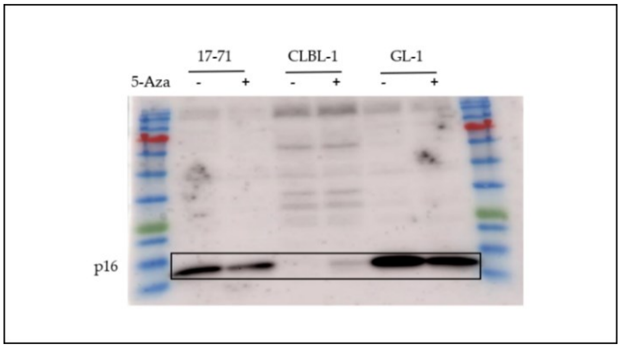

(b)

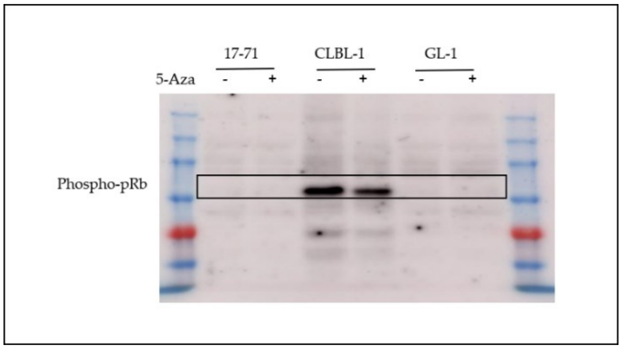

(c)

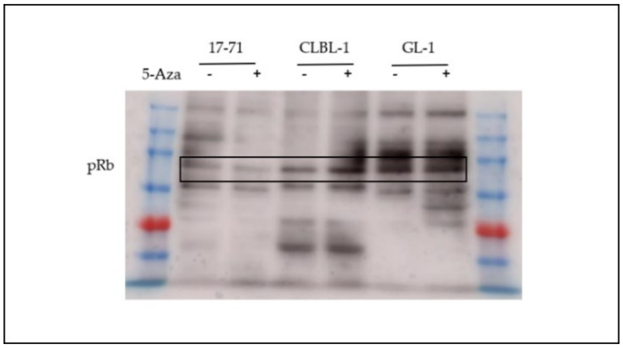

(d)

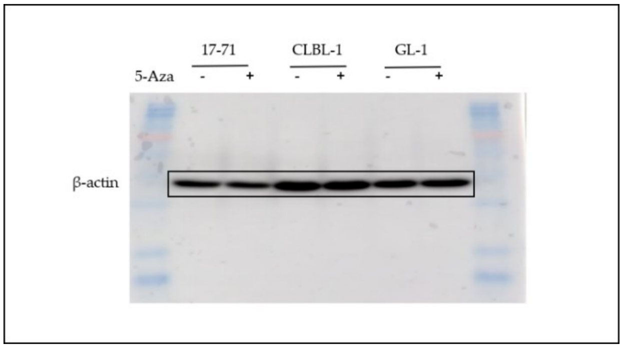

Figure S4. Original images for Figure 3. The expression of the p16 protein in canine lymphoma and leukemia B-cell lines treated with or without 5-Aza (a); phospho-pRb (b); pRb (c); and β-actin as endogenous control (d). The protein expression was assessed using the western blot analysis.

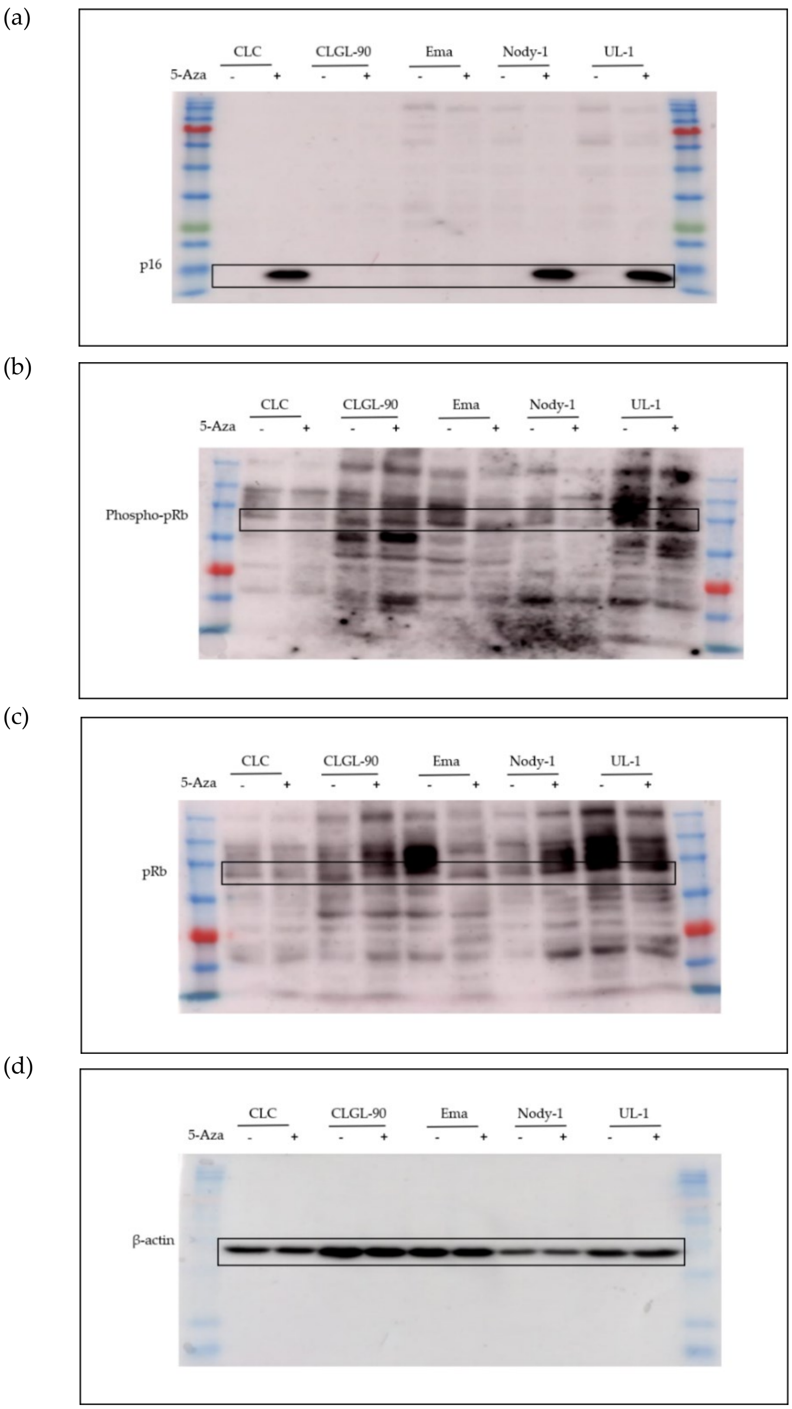

Figure S5. Original images for Figure 4. The expression of the p16 protein in canine lymphoma and leukemia T-cell lines treated with or without 5-Aza (a); phospho-pRb (b); pRb (c); and β-actin as endogenous control (d). The protein expression was assessed using the western blot analysis.
